# Conditional Inactivation of Limbic Neuropeptide Y-1 Receptors Increases Vulnerability to Diet-Induced Obesity in Male Mice

**DOI:** 10.3390/ijms22168745

**Published:** 2021-08-14

**Authors:** Silvia Paterlini, Riccardo Panelli, Laura Gioiosa, Stefano Parmigiani, Paolo Franceschini, Ilaria Bertocchi, Alessandra Oberto, Alessandro Bartolomucci, Carola Eva, Paola Palanza

**Affiliations:** 1 Unit of Neuroscience, Department of Medicine and Surgery, University of Parma, 43100 Parma, Italy; riccardopanelli@gmail.com (R.P.); laura.gioiosa@unipr.it (L.G.); paolof1980@libero.it (P.F.); 2 Department of Chemistry, Life Sciences and Environmental Sustainability, University of Parma, 43100 Parma, Italy; stefano.parmigiani@unipr.it; 3 Neuroscience Institute of the Cavalieri-Ottolenghi Foundation, 10043 Orbassano, Italy; ilaria.bertocchi@unito.it (I.B.); alessandra.oberto@unito.it (A.O.); carola.eva@unito.it (C.E.); 4 Department of Neuroscience, University of Turin, 10126 Turin, Italy; 5 Department of Physiology, University of Minnesota, Minneapolis, MN 55455, USA; abartolo@umn.edu

**Keywords:** NPY, Y1R, high fat diet, obesity, glucose intolerance, food intake

## Abstract

NPY and its Y1 cognate receptor (Y1R) have been shown to be involved in the regulation of stress, anxiety, depression and energy homeostasis. We previously demonstrated that conditional knockout of *Npy1r* gene in the excitatory neurons of the forebrain of adolescent male mice (Npy1r*^rfb^* mice) decreased body weight growth and adipose tissue and increased anxiety. In the present study, we used the same conditional system to examine whether the targeted disruption of the *Npy1r* gene in limbic areas might affect susceptibility to obesity and associated disorders during adulthood in response to a 3-week high-fat diet (HFD) regimen. We demonstrated that following HFD exposure, Npy1r*^rfb^* male mice showed increased body weight, visceral adipose tissue, and blood glucose levels, hyperphagia and a dysregulation of calory intake as compared to control Npy1r*^2lox^* mice. These results suggest that low expression of *Npy1r* in limbic areas impairs habituation to high caloric food and causes high susceptibility to diet-induced obesity and glucose intolerance in male mice, uncovering a specific contribution of the limbic *Npy1r* gene in the dysregulation of the eating/satiety balance.

## 1. Introduction

Neuropeptide Y (NPY) is a widely distributed peptide that is involved in the regulation of several biological functions including energy balance, emotional and stress reactions, and cognition [1,2,3,4]. Injection of NPY into either the cerebral ventricles or the hypothalamus stimulates food intake even in satiated rats, eventually leading to obesity [5,6]. In addition, NPY is a stress-activated sympathetic cardiovascular and metabolic regulator that could influence co-morbidity patterns of stress-related disorders such as the metabolic syndrome [7].

Dysfunctions of the NPY system have been implicated in diseases such as obesity, type II diabetes and metabolic syndrome. Obesogenic diets alter the hypothalamic NPY expression (reviewed in [8]), and elevated levels of NPY gene expression are found in several models of animal obesity such as diet-induced obesity (DIO) [9], leptin deficient *ob/ob* mice [10,11] and Zucker fatty *fa/fa* rats [12]. NPY release is also elevated in the obese state, as shown in the Zucker rat [13], and pharmacological treatments that enhance feeding also increase NPY gene expression [14]. In humans, one variant causing an amino acid change from leucine to proline at codon 7 in the signal peptide of NPY alters the packaging and secretion of NPY, leading to an increased peptide synthesis and release in endocrine cells and central nervous system (CNS) neurons [15]. Consistently, Leu7/Pro polymorphism of NPY gene is associated with increased body mass index [16], impaired glucose tolerance and type II diabetes [17,18], higher serum levels of total and low-density lipoprotein cholesterol [19], and predicts myocardial infarction and stroke in hypertensive patients [20]. Moreover, elevated plasma levels of NPY during exercise and elevated sympathetic activity was observed in subjects carrying the NPY Leu7/Pro polymorphism [21]. Taken together, these findings suggest that an increased NPYergic tone leading to an enhanced NPY production may be an etiological factor in metabolic syndrome.

In the brain, NPY interacts with a family of G protein–coupled receptors that includes the Y1 (Y1R), Y5 and Y2 receptors [2]. Numerous studies suggest that NPY affects metabolism and feeding behavior mainly by the activation of the Y1R. The role of hypothalamic Y1R in mediating NPY-induced hyperphagia has been an early focus in obesity research [1,2,22,23]. Further studies revealed that, in addition to feeding, Y1R mediates other metabolic effects of NPY related to energy homeostasis, including a decrease in energy expenditure and lipid metabolism and an increase of insulin secretion [24,25,26,27].

We previously showed that conditional inactivation of *Npy1r* gene in the forebrain excitatory neurons of adolescent mice reduced body weight, adipose tissue, and serum leptin levels in males (Npy1r*^rfb^* mice, rfb = reduced forebrain; [28,29]). Moreover, Npy1r*^rfb^* male mice had higher peripheral corticosterone, density of NPY immunoreactive fibers and corticotropin releasing hormone (CRH) immunoreactive cell bodies in the hypothalamic paraventricular nucleus (PVN) compared to their control littermates (Npy1r*^2lox^* mice), suggesting that conditional inactivation of limbic *Npy1r* might decrease body weight gain by activation of the hypothalamic–pituitary–adrenal (HPA) axis.

We have previously shown that limbic *Npy1r* expression is regulated by early maternal environment and it might be a key target of maternal care-induced programming of energy homeostasis [28]. Differences in the phenotype between Npy1r*^rfb^* and Npy1r*^2lox^* mice only became apparent when both genotypes were raised by foster dams exhibiting high levels of arched back nursing (HABN), an index of high maternal (HM) care towards their adopted pups [30,31]. 

In the current study we examined whether the targeted disruption of *Npy1r* gene in excitatory neurons of the limbic system of adolescent mice might affect susceptibility to obesity and associated disorders in response to metabolic challenges. To this purpose, we exposed Npy1r*^rfb^* and Npy1r*^2lox^* mice raised by HM dams to a high fat diet for 3 weeks and measured metabolic and neuroendocrine functions and markers of type 2 diabetes (see “Materials and Methods” section or experimental timeline).

## 2. Results

### 2.1. Analysis of Spontaneous Maternal Behavior of Swiss CD1 Foster Dams

**Figure 1:**
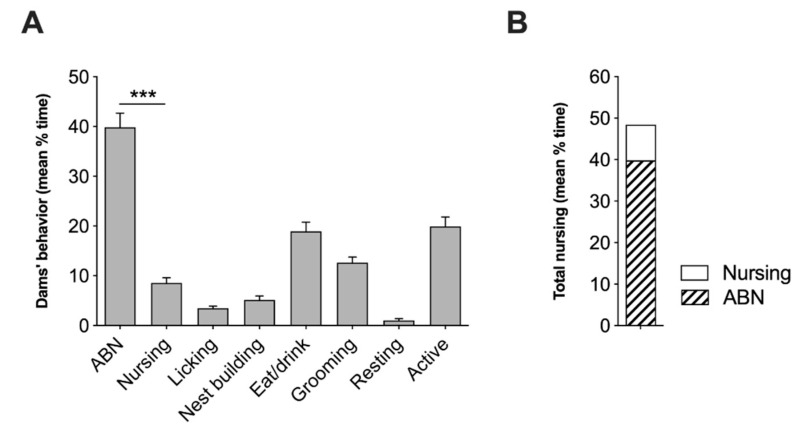
Spontaneous maternal behavior of CD1 foster dams. (**A**) Average percent time spent by CD1 foster dams in different behaviors and (**B**) total nursing during postnatal day 1–7. Data are presented as mean ±SEM. Statistical analysis was performed by using Wilcoxon signed rack test. *** *p* < 0.001.

Spontaneous maternal behavior of Swiss CD1 foster dams was observed throughout the first week of postnatal life. In Figure 1A, we showed the mean percentage of time spent in maternal behavior obtained by direct observation of mother-pups interaction. CD1 foster dams spent most of the time engaged in pup-related behaviors by crouching over the pups in the active form of nursing known as arched-back nursing (ABN) compared to the lying posture of nursing pups (Figure 1A,B) (*z* = 3.82, *p* < 0.001). The behavioral profile of CD1 foster mothers indicates high levels of maternal care.

### 2.2. Body Weight Growth

**Figure 2:**
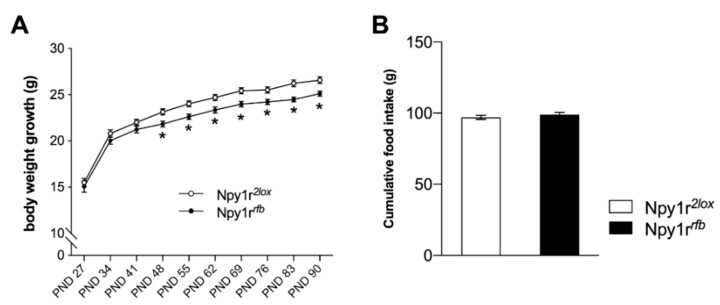
Body weight and food intake of standard diet (SD)-fed control and Npy1r*^rfb^* knockout male mice. (**A**) Body weight growth and (**B**) cumulative food intake during PND 27–90 of Npy1r*^2lox^* and Npy1r*^rfb^* male mice reared by CD1 foster dams. Data are presented as mean ± SEM. * *p* < 0.05. PND, post-natal day.

From weaning to adulthood, we monitored body weight growth in Npy1r*^rfb^* and Npy1r*^2lox^* male mice reared by CD1 dams. Consistent with our previous studies [28,29], Npy1r*^rfb^* mice are characterized by a lower body weight growth starting from postnatal day (PND) 48 (when total inactivation of *Npy1r* gene occurs) as compared to control Npy1r*^2lox^* male mice (Figure 2A; genotype by days interaction: F_(9,324)_ = 1.91; *p* = 0.049). According to previous studies [32], food intake (Figure 2B) and locomotor activity (data not shown) did not significantly differ between genotypes.

### 2.3. Metabolic Consequences of Diet Exposure

#### 2.3.1. Body Weight and Food Intake

**Figure 3:**
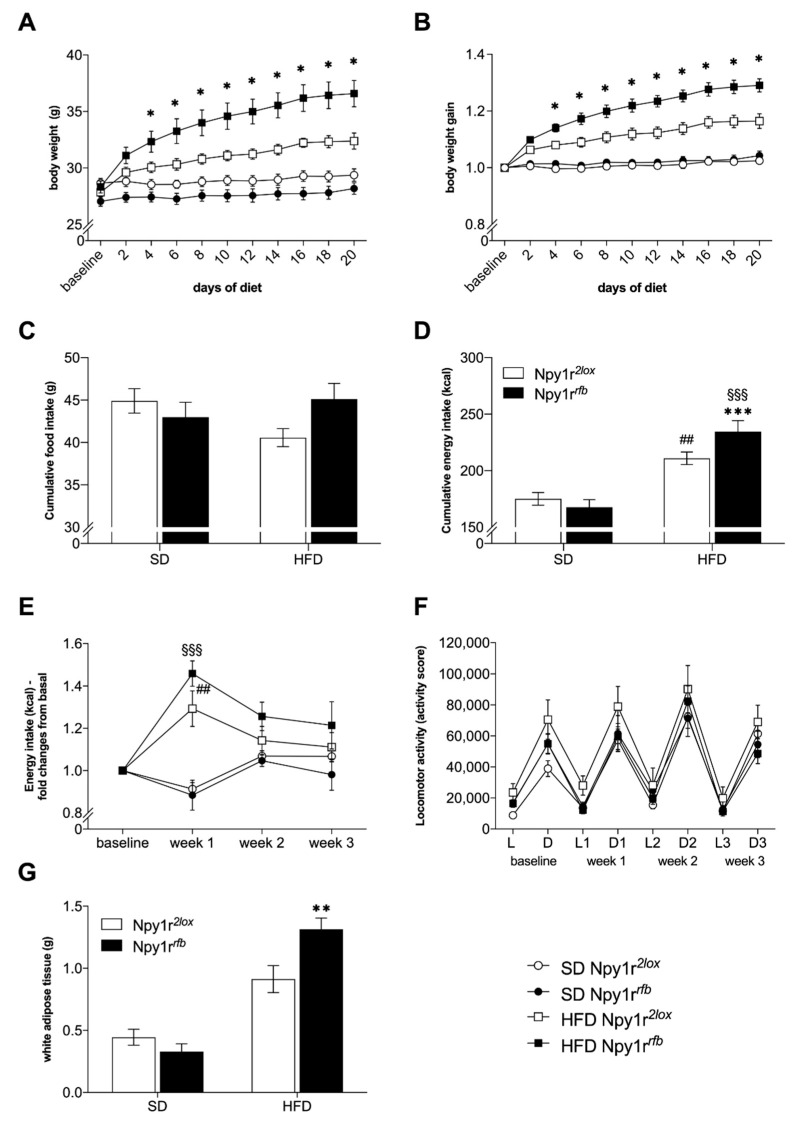
Metabolic consequences of HFD in Npy1r*^2lox^* and Npy1r*^rfb^* male mice. (**A**) Body weight growth, (**B**) body weight gain, (**C**) cumulative food intake (g), (**D**) cumulative energy (kcal) intake and (**E**) weekly energy intake (kcal) of SD- and HFD-fed Npy1r*^2lox^* and Npy1r*^rfb^* mice during the metabolic challenge procedure. (**F**) Spontaneous locomotor activity of Npy1r*^2lox^* and Npy1r*^rfb^* male mice exposed to SD or HFD for 21 days. (**G**) White adipose tissue weight (g) of Npy1r*^2lox^* and Npy1r*^rfb^* male mice exposed to SD or HFD for 26 days. Data are presented as mean ± SEM. Food intake was calculated as means of the sum of grams for mouse (**C**); energy intake was calculated as means of the sum of kilocalories (**D**) for mouse and as fold changes versus baseline (**E**). (**A**,**B**) * *p* < 0.05 versus Npy1r*^2lox^* on HFD; (**D**) *** *p* < 0.001 versus Npy1r*^2lox^* on HFD, ^##^ *p* < 0.01 versus Npy1r*^2lox^* on SD, ^§§§^ *p* < 0.001 versus Npy1r*^rfb^* on SD; (**E**) ^##^ *p* < 0.01 versus Npy1r*^2lox^* on SD, ^§§§^ *p* < 0.001 versus Npy1r*^rfb^* on SD; (**G**) ** *p* < 0.01 versus Npy1r*^2lox^* on HFD.

Overall, high fat diet (HFD) resulted in a significant increase of mice body weight growth compared to standard diet (SD), independently of the genotype (Figure 3A; diet: F_(1,36)_ = 46.294; *p* < 0.001). Starting from the fourth day of HFD exposure, Npy1r*^rfb^* males showed a significantly greater body weight (Figure 3A; diet by genotype by days interaction: F_(10,360)_ = 5.834; *p* < 0.001) and a significant greater body weight gain (Figure 3B; significant diet by genotype by days interaction: F_(9,324)_ = 3.94; *p* < 0.001) as compared to Npy1r*^2lox^* males, whereas no significant differences were observed between SD-fed Npy1r*^rfb^* and Npy1r*^2lox^* mice.

Analysis of feeding behavior showed that, over the 3 weeks of SD regimen, both Npy1r*^rfb^* and Npy1r*^2lox^* mice consumed the same total amount of food (Figure 3C). Conversely, when exposed to HFD, Npy1r*^2lox^* mice reduced the total amount of food intake, whereas HFD-fed Npy1r*^rfb^* mice did not (Figure 3C; genotype by diet interaction: F_(1,36)_ = 4.609, *p* = 0.039). Accordingly, although exposure to HFD increased total energy intake in both genotypes (Figure 3D; diet: F_(1,36)_ = 55,386, *p* < 0.0001), the cumulative energy intake of HFD-fed Npy1r*^rfb^* mice was significantly higher than that measured in their control littermates on the same diet regimen (Figure 3D; genotype by diet interaction: F_(1,36)_ = 5.065, *p* = 0.031, Npy1r*^rfb^* HFD vs. Npy1r*^2lox^* HFD: *p* < 0.001). 

These data are confirmed by the temporal analysis of energy intake in Npy1r*^rfb^* and Npy1r*^2lox^* mice. Both genotypes increased energy intake during the first week of HFD and they both reduced it on the 2nd and 3rd week of HFD exposure (Figure 3E; week by diet interaction: F_(2,72)_ = 19,220; *p* < 0.001). However, while control Npy1r*^2lox^* mice showed a complete adjustment of kcal intake during the 2nd and 3rd week of HFD exposure, Npy1r*^rfb^* mice on HFD continued to display higher kcal intake than Npy1r*^rfb^* mice on SD (Figure 3E). 

#### 2.3.2. Locomotor Activity

Locomotor activity was monitored throughout the whole experiment including the 1-week baseline phase and the following 21 days of metabolic challenge. Statistical analysis did not show any significant difference in locomotor activity during the light nor the dark phase among SD- and HFD-fed Npy1r*^2lox^* and Npy1r*^rfb^* male mice independently of the diet (Figure 3F). 

#### 2.3.3. Visceral Adiposity

An increase in the visceral fat weight (perigonadal and mesenteric fat tissue) was observed in both Npy1r*^2lox^* and Npy1r*^rfb^* mice exposed to HFD, as compared to mice on SD (Figure 3G; diet: F_(1,36)_ = 65.46; *p* < 0.001). Significantly larger depots of visceral fat in HFD-fed Npy1r*^rfb^* mice compared to HFD-fed Npy1r*^2lox^* mice were also observed (*p* = 0.013), while Npy1r*^rfb^* and Npy1r*^2lox^* mice on SD had similar visceral adiposity (Figure 3G; diet by genotype interaction: F_(1,36)_ = 8.27; *p* < 0.01).

#### 2.3.4. Blood Glucose Level 

**Figure 4:**
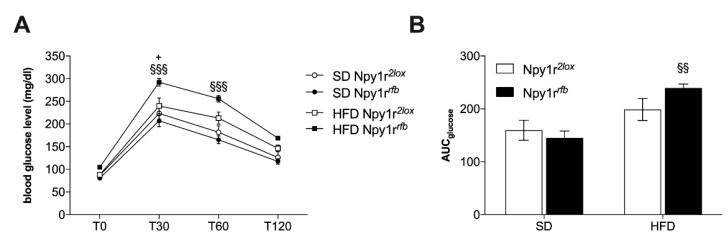
(**A**) Glucose tolerance test and (**B**) area under the curve (AUC) of Npy1r*^2lox^* and Npy1r*^rfb^* male mice 21 days after SD or HFD exposure. Data are presented as mean ± SEM. (**A**) ^+^ *p* = 0.067 versus Npy1r*^2lox^* on HFD, ^§§§^ *p* < 0.001 versus Npy1r*^rfb^* on SD; (**B**) ^§§^ *p* < 0.01 versus Npy1r*^rfb^* on SD.

Analysis of glucose tolerance showed that HFD promoted glucose intolerance in Npy1r*^rfb^* males compared to Npy1r*^2lox^* males (Figure 4A; diet by genotype interaction F_(1,36)_ = 7.11, *p* = 0.011). Moreover, Npy1r*^rfb^* mice on HFD showed significantly higher area under the curve (AUC) as compared to Npy1r*^rfb^* mice on SD (Figure 4B; *p* = 0.0067).

### 2.4. Plasma Analysis

Plasma levels of total cholesterol, HDL, LDL and triglycerides are reported and summarized in Table 1. In both Npy1r*^2lox^* and Npy1r*^rfb^* male mice, HFD significantly increased plasma levels of total cholesterol (diet: F_(1,28)_ = 42,755, *p* < 0.001), HDL (diet: F_(1,28)_ = 22,810, *p* < 0.001) and LDL (diet: F_(1,28)_ = 19,5879, *p* < 0.001), whereas no differences due to HFD on plasma triglycerides levels were observed. No significant difference in these parameters were observed between Npy1r*^2lox^* and Npy1r*^rfb^* independently of the diet.

**Figure 5:**
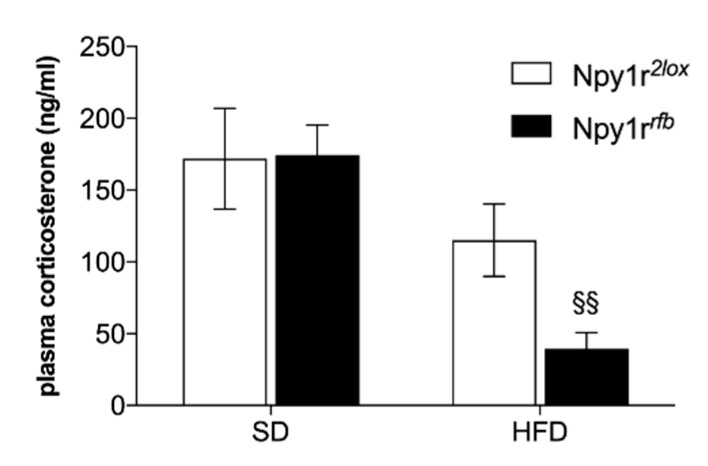
Corticosterone levels. Npy1r*^rfb^* mice on HFD showed a significant reduction of corticosterone plasma levels compared to the SD-fed group. Values are presented as mean ± SEM. ^§§^ *p* < 0.01 versus Npy1r*^rfb^* on SD.

Mice of both genotypes fed HFD also showed a significant reduction of plasma corticosterone as compared to mice on SD (Figure 5; diet: F_(1,16)_ = 15.17, *p* < 0.01). This difference is particularly pronounced in Npy1r*^rfb^* mice on HFD, which showed a significantly lower corticosterone plasma levels compared to SD-fed Npy1r*^rfb^* mice (*p* < 0.01 by Tukey). 

### 2.5. Npy1r mRNA Expression

**Figure 6:**
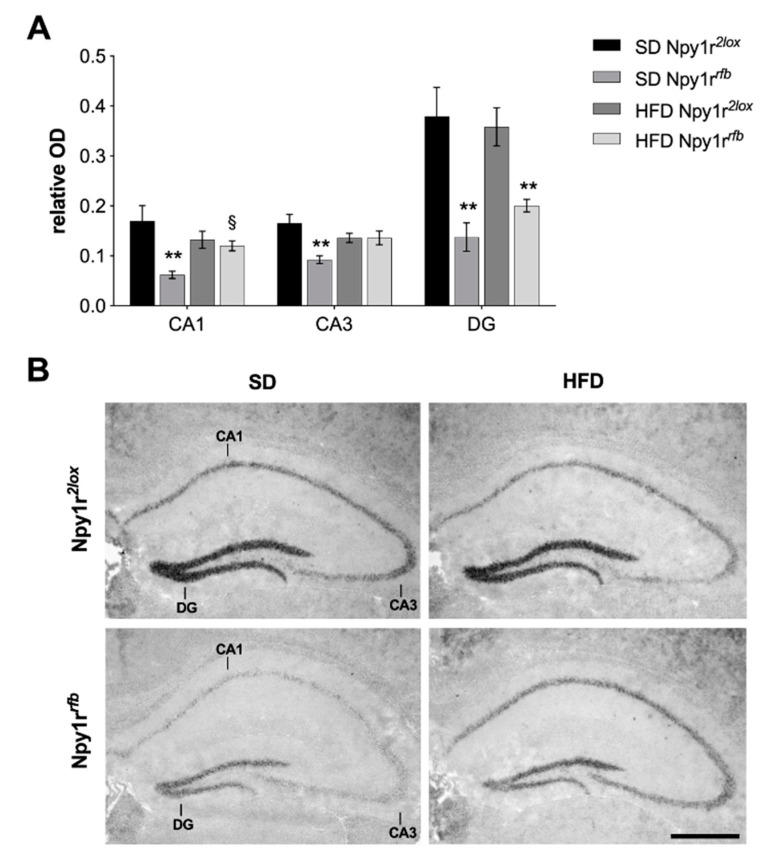
*Npy1r* mRNA expression in the hippocampus of Npy1r*^2lox^* and Npy1r*^rfb^* mice fed with SD and HFD. (**A**) *Npy1r* mRNA expression in the hippocampus of Npy1r*^2lox^* and Npy1r*^rfb^* mice fed with SD and HFD. (**B**) Representative pictures showing *Npy1r* mRNA signal intensity differences in the CA1, CA3 and DG of SD- and HFD-fed Npy1r*^2lox^* and Npy1r*^rfb^* male mice. Scale bars: 250 μm. CA1, cornu ammonis 1 hippocampal subregion; CA3, cornu ammonis 3 hippocampal subregion; DG, dentate gyrus. Values are presented as mean ± SEM (**A**). ** *p* < 0.01 versus Npy1r*^2lox^* mice. ^§^ *p* < 0.05 versus Npy1r*^rfb^* mice on SD. Scale bars: 500 µm.

Conditional Cre-mediated inactivation of *Npy1r* gene was verified by semiquantitative in situ hybridization. As pictured in Figure 6, Npy1r*^rfb^* males on SD showed a significantly lower *Npy1r* mRNA expression in cornu ammonis 1 (CA1) (genotype by diet interaction: F_(1,25)_ = 5.97, *p* = 0.022; Tukey: *p* < 0.01), cornu ammonis 3 (CA3) (genotype by diet interaction: F_(1,25)_ = 5.59, *p* = 0.026; Tukey: *p* = 0.04) and dentate gyrus (DG) (genotype: F_(1,25)_ = 35.41, *p* < 0.001; Tukey: *p* < 0.001) subregions of the hippocampus compared to Npy1r*^2lox^* control littermates, as previously reported [28].

**Figure 7:**
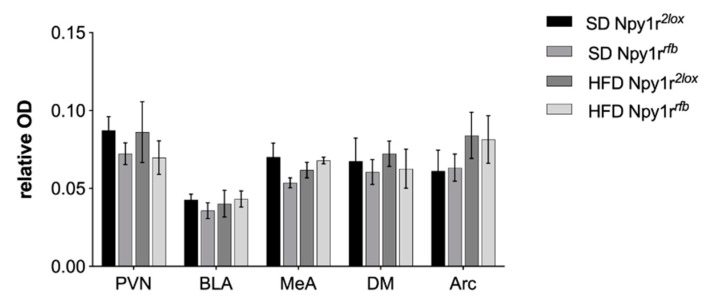
Relative optic densities (OD) of *Npy1r* mRNA expression in the hypothalamus and amygdala of Npy1r*^2lox^* and Npy1r*^rfb^* mice fed with SD and HFD. PVN, paraventricular hypothalamic nucleus; DM, dorsomedial hypothalamic nucleus; ARC, arcuate hypothalamic nucleus; BLA, basolateral amygdala; MeA, medial amygdala.

Npy1r*^rfb^* mice exposed to HFD showed increased *Npy1r* mRNA in the CA1 (genotype by diet interaction: F_(1,25)_ = 5.97, *p* = 0.022) and in the CA3 (genotype by diet interaction: F_(1,25)_ = 5.59, *p* = 0.026), compared to Npy1r*^rfb^* mice on SD. Accordingly, when exposed to HFD, Npy1r*^rfb^* males showed lower *Npy1r* mRNA expression in the DG (genotype: F_(1,25)_ = 35.41, *p* < 0.001; Tukey: *p* < 0.01), but not in CA1 and CA3, when compared to Npy1r*^2lox^* control mice (Figure 5). No significant *Npy1r* mRNA expression differences were observed in the paraventricular (PVN), dorsomedial (DM) and arcuate (Arc) hypothalamic nuclei and in the basolateral (BLA) and medial (MeA) amygdala of Npy1r*^rfb^* and Npy1r*^2lox^* mice fed with SD or HFD (Figure 7).

## 3. Discussion

In this study we investigated the effects of limbic Y1R on vulnerability to diet-induced obesity and associated disorders that may be indexes of metabolic syndrome, i.e., a collection of metabolic risk factors that includes obesity, insulin resistance and dyslipidemia [33]. To this aim, we used a Npy1r*^rfb^* mouse model in which the postnatal conditional inactivation of *Npy1r* gene in hippocampal excitatory neurons decreases body weight growth and adipose tissue on normal standard diet regimen [28]. Here, we demonstrated that mutant male mice exposed to HFD showed increased caloric intake, body weight growth, abdominal adipose tissue and blood glucose levels compared to their control littermates, suggesting increased vulnerability of Npy1r*^rfb^* male mice to metabolic challenge.

Bertocchi et al. [28] previously showed that the altered metabolic functions of Npy1r*^rfb^* mice can be observed only when mice are reared by FVB/J foster mothers displaying high maternal care. In the present study, Npy1r*^rfb^* and Npy1r*^2lox^* mice were fostered to Dox-naïve Swiss CD1 dams showing a high maternal care profile similar to the FVB/J maternal behavior previously described [28]. Consistently, Npy1r*^rfb^* males reared by CD1 dams were characterized by low hippocampal *Npy1r* mRNA expression and slower body weight growth after PND 40, which coincides with the maximal levels of *Npy1r* gene Cre-mediated inactivation.

Exposure to HFD increased body weight growth, visceral fat and plasma cholesterol, LDL and HDL in both Npy1r*^rfb^* and Npy1r*^2lox^* male mice compared with SD-fed groups, yet HFD more profoundly altered the metabolic functions of Npy1r*^rfb^* mice compared to control Npy1r*^2lox^* mice. HFD-fed Npy1r*^rfb^* males showed a significant increase in body weight gain, abdominal fat weight and a slower glucose clearance compared to HFD-fed Npy1r*^2lox^* controls. This greater increase in body weight was observed four days after HFD exposure and persisted throughout the remaining 21 days of the diet regimen.

Different factors could contribute to the increased vulnerability of Npy1r*^rfb^* male mice to metabolic challenges. Although no detailed information on energy expenditure was obtained, the effects of HFD exposure on body weight growth, visceral fat and glycaemia of Npy1r*^rfb^* mice cannot be ascribed to differences in locomotor activity, which was similar in both genotypes either during the dark or the light phase.

Conversely, across the 3 weeks of diet exposure, Npy1r*^rfb^* and Npy1r*^2lox^* mice showed a different adjustment to HFD intake. While control Npy1r*^2lox^* mice showed reduced HFD consumption as compared to Npy1r*^2lox^* mice on SD, HFD-fed Npy1r*^rfb^* mutant mice showed hyperphagia, particularly during the first week of HFD exposure, as compared to SD-fed Npy1r*^rfb^* mice. As a result, even though HFD increased energy intake in both genotypes compared to SD, the total energy intake over the three weeks of HFD regimen was significantly higher in Npy1r*^rfb^* mice than in Npy1r*^2lox^* control mice. Thus, the greater body weight gain observed in Npy1r*^rfb^* males on HFD might be ascribed to an impaired adjustment to HFD intake that results in higher kcal intake during the whole period of diet as compared to Npy1r*^2lox^* mice.

A complex interplay between mood, emotional state and eating behavior is recognized to influence body weight and susceptibility to metabolic disorders (e.g., obesity) [34,35]. Humans and animals show increased preference for highly palatable food (“comfort food”) during stress and a subsequent accumulation of visceral fat, both of which have been ascribed to the higher plasmatic stress hormone concentration [36]. At the same time, it has been suggested that eating “comfort” food can reduce the activity of the chronic stress-response network; indeed, ingestion of highly palatable food decreases CRF mRNA expression in the hypothalamus of rats, attenuating the stress response [34]. Although CD1 fostered Npy1r*^rfb^* mice in SD do not display increased HPA axis activity, preliminary results obtained in a different cohort of animals (Paterlini et al. in preparation) indicate that they show an anxious-like behavior, as also previously reported [28,29]. Thus, the greater ingestion of HFD displayed by Npy1r*^rfb^* mice could be ascribed to their higher anxiety levels and could account for both the increased body weight growth and the reduction of plasmatic corticosteroid levels. The observed hyperphagia associated to an impaired habituation to high caloric food in NPY1r*^rfb^* mice might suggest that limbic NPY-Y1R system play a role in emotional eating disorder phenotype resembling human binge eating associated with obesity. Although this is a speculative hypothesis, here is evidence that emotional eating is involved in the development and maintenance of obesity and binge eating, characterized by the consumption of a large amount of high caloric food in brief periods of time and by emotional alterations [37,38,39,40,41].

On the other hand, here we observed that the exposure of Npy1r*^rfb^* mice to HFD increased *Npy1r* mRNA in the CA1 and CA3, thus abolishing the difference in *Npy1r* mRNA expression between control and mutant mice in these hippocampal regions.

In our opinion, this effect cannot be ascribed to a failure of the *Npy1r* gene deletion in the mutant mice used in this study since, when grown in standard conditions, Npy1r*^rfb^* mice showed lower body weight growth than control Npy1r*^2lox^* mice, therefore displaying a phenotype consistent with the conditional deletion of the *Npy1r* gene [28]. Moreover, a significant decrease of *Npy1r* mRNA was found in the DG of HFD-fed Npy1r*^rfb^* mice as well as in the CA1, CA3 and DG of their SD-fed mutant littermates. Although the mechanism by which HFD increased *Npy1r* mRNA expression only in the CA1 and CA3 of Npy1r*^rfb^* conditional knockout mice is unknown, it could reflect a compensatory response to a metabolic challenge that might be at least in part responsible for their higher predisposition to develop diet-induced obesity and glucose intolerance.

Given that Npy1r*^rfb^* mice carry the conditional inactivation of *Npy1r* gene selectively in principal neurons, we suggest that the increase of *Npy1r* mRNA in HFD-fed mutant mice could occur in non-glutamatergic cells of the CA1 and CA3 regions. This is in line with the observation that, in the hippocampus, Y1Rs are expressed not only in principal neurons, but also in NPY positive neurons as well as in glial cells [42,43,44]. Accordingly, the inactivation of *Npy1r* gene in excitatory neurons of Npy1r*^rfb^* mice reduces, but does not completely deplete, *Npy1r* gene expression in the hippocampus (see present results and [28]). 

On the other hand, we previously demonstrated that CD1 fostered male Npy1r*^rfb^* mice show a phenotype characterized by decreased vagal modulation and higher sympathetic activity [32]. It is well known that chronic activation of the sympathetic system, that induces beta receptor downregulation and failure of energy dissipation, is a predictive factor for visceral adiposity and metabolic syndrome [45]. Accordingly, mice lacking beta receptors (β-less mice) develop severe obesity following a HFD regimen because of a failure of diet-induced thermogenesis and cathecolamine-mediated lipolysis in WAT [46]. Thus, we cannot exclude that the increased sympathetic activity of CD1-fostered Npy1r*^rfb^* male mice might also account for their increased vulnerability to diet induced metabolic disorders.

In conclusion, here we provided the first demonstration that the inactivation of limbic *Npy1r* gene increases susceptibility to diet-induced obesity and glucose intolerance in male mice that can be due to a dysregulation of the appetite/satiety balance. We suggest that the conditional inactivation of limbic *Npy1r* gene in Npy1r*^rfb^* mice might mimic, at least in part, the mechanisms underlying emotional eating, such as binge eating disorders and associated obesity, a hypothesis that needs further investigations.

## 4. Materials and Methods

### 4.1. Experimental Outline

**Figure 8:**
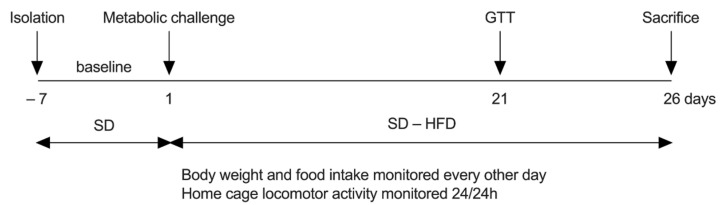
Experimental outline.

Adult male mice carrying the conditional inactivation of *Npy1r* gene (Npy1r*^rfb^*) in the forebrain, and their control siblings (Npy1r*^2lox^*) were fed a hypercaloric, high fat diet (HFD) or a standard diet (SD) for 26 days. Body weight, food intake and locomotor activity were determined throughout the experiment. On day 21 of the diet regimen, mice underwent a glucose tolerance test (GTT). At the end of the diet regimen, visceral adipose tissue, plasma and brains were collected and analyzed, as described below (Figure 8). 

### 4.2. Animals and Housing

All mice used were born and reared in the laboratory of Behavioral Biology at the University of Parma and housed in a temperature—(22 ± 1 °C) and humidity—(60 ± 10%) controlled room on a 12-h light/dark cycle with ad libitum access to food (Mucedola, Milan, Italy—4RF21 standard diet) and water, and to sunflower seeds (Mundi, Siena, Italy) in addition to normal food, for nutritional and environmental enrichment. 

The generation of Npy1r*^2lox^* and Npy1r*^rfb^* B6129S (129/SvJ, C57BL/6N derived strain) mice was achieved as previously described [28]. Briefly, a targeting vector for homologous recombination in embryonic stem (ES) cells was designed to introduce *loxP* sites around exons 2–3, which code for the entire region of *Npy1r*, and a *frt-neo-frt* cassette ≈450 bp excised from p146 (kind gift of William Wisden) upstream the ATG of *Npy1r* exon 2. The linearized targeting vector was electroporated into 5 × 107 R1 ES cells (129Sv × 129sv − CP) [47] to create a conditional *Npy1r* allele (*Npy1r^+/loxP−neo^*), containing two *loxP* sites and a *frt* flanked neomycin selection cassette. R1–ES cells of a correctly targeted clone for the recombination (*Npy1r^+/loxP−neo^*) were then expanded for injection into C57BL/6N blastocysts that were implanted into pseudopregnant ICR female recipients. We obtained 11 chimeric founders and three chimeric males transmitted the R1–ES cells and produced offspring with agouti coat color. PCR and Southern blot analysis of tail DNA revealed that 50% of the pups contained the gene targeted *Npy1r* allele. Subsequently, the *neo* gene was removed by flp-mediated recombination in vivo, by crossing *Npy1r^+/loxP−neo^* offspring with *flp* expressing C57BL/6N FLP deleter mice [48]. To obtain mice with *Npy1r* gene inactivation restricted to excitatory neurons (Npy1r*^rfb^* mice) three different mouse lines [Npy1r*^2lox^*, Tgα*^CamKII−tTA^* [49] and Tg*^LC1^* [50]] underwent specific breeding protocol: we generated (i) a Npy1r*^2lox^*/Tgα*^CamKII−tTA^* mouse line, expressing the tTA in a cell type-specific manner under the control of the αCamKII promoter and (ii) a Npy1r*^2lox^*/Tg*^LC1^* line, encoding the tTA-responsive Cre transgene TgLC1. Region-specific inactivation of the *Npy1r* gene was achieved by crossing Npy1r*^2lox^*/Tgα*^CamKII−tTA^* with Npy1r*^2lox^*/Tg*^LC1^* mice. Doxycycline treatment of mothers (50 mg/L in drinking water, 1% sucrose) from conception to birth keeps the Cre transgene inactive in embryos and synchronizes the postnatal Cre activation. On P0, pups were moved to Dox naïve foster mothers. In Npy1r*^2lox^*/Tgα*^CamKII−tTA^*/Tg*^LC1^* mice (Npy1r*^rfb^* mice) Cre expression was fully activated at approximately postnatal day (PND) 40 [28]. 

Npy1r*^2lox^* and Npy1r*^rfb^* mice were maintained in a randomly segregating population derived from more than 500 different breeding pairs and each litter provided the same number of experimental mice to both Npy1r*^2lox^* and Npy1r*^rfb^* groups. Npy1r*^2lox^* control mice did not show any recombination of the floxed *Npy1r* allele and were used as control littermates in all experiments [51].

Tgα*^CamKII−tTA^* mice were backcrossed for more than 10 generations to C57Bl/6N mice, while the other mouse lines were bred in their colony; the coat color of the investigated mice was still mixed ‘mainly black’, indicating that the genetic background of the investigated population is very heterogeneous, which suggests that the observed phenotype is independent from the genetic background [52]. Genotyping was performed as described previously [28,53].

On P0, pups were moved to Dox naïve CD1 foster mothers whose spontaneous maternal behavior was observed from PND 1 to 7, as described below, and maintained on a 11 a.m.:11 p.m. 12-h light/dark cycle. On PND 27, mice were weighed, individually marked with an ear tag and housed in same-sex groups of siblings (2–5 per cage) in Plexiglas cages (45 cm * 25 cm * 20 cm) with wooden dust-free bedding (Scobis, Mucedola, Milan, Italy) and paper for nest-building changed weekly. Starting from weaning, the 12-h light/dark cycle was shifted one hour a week to a 9 a.m.:9 p.m. light cycle. 

### 4.3. Maternal Behavior

On PND 1–7, spontaneous maternal behavior was assessed by observing lactating females (CD1 foster dams *n* = 19) in their home cages with wooden dust-free bedding (Scobis, Mucedola, Milan, Italy) and paper for nest-building changed weekly. Mice are mostly active at night, thus the observations were conducted in the last two hours of the dark phase (9–11 a.m.) with the aid of 25-W red lights. By means of an instantaneous sampling, each dam was observed once every 4 min for a total of 30 observations. During each 4-min observation period, the experimenter recorded which behavior the lactating female was displaying following the behavioral categories as described in Palanza et al. [54], namely: Arched back nursing: the female was nursing pups with her body arched over them; In nest: the female was anywhere inside the nest, regardless of the behavior being exhibited at the moment of observation; Nursing: the female was allowing the pups to suckle; this category did not necessarily imply that the whole litter was nursing; Licking pups: the female was licking or grooming her pups; Nest building: the female was engaged in some aspect of nest building, while she was either inside or outside the nest itself; Eating/drinking: the female was nibbling at a food pellet or drinking from the water bottle; Grooming: The female was grooming her own body; Active: The female was moving in the cage; Resting: The female was lying motionless outside the nest, not involved in any other form of behavior and with no pup attached to her nipples; Out of nest: the female was outside the nest.

### 4.4. Body Weight Growth

Offspring body weight was monitored from birth to adulthood by a digital balance accurate to 0.01 g (Sartorius, Goettingen, Germany) with the animal weighing functions (animal’s weight is automatically calculated as the average of a defined number of individual weighing operations). Pups were weighed immediately after birth (PND 0), at PND 10 and at weaning (PND 27). From weaning to the beginning of the diet exposure procedure, body weight was measured once a week from 10 a.m. to 11 a.m. to check body weight growth. At PND 113, male mice were individually housed in Plexiglas cages (45 cm × 25 cm × 20 cm) with wooden dust-free bedding (Scobis, Mucedola, Milan, Italy) and paper for nest-building changed weekly. Only males were used in the present study; their female siblings were used in a different parallel study that we plan to submit in a subsequent manuscript.

### 4.5. Diet Exposure

On PND 120, male mice on a standard diet were subjected to a baseline recording of body weight, food intake and locomotor activity. After a week, Npy1r*^rfb^* and Npy1r*^2lox^* mice, which were matched as much as possible for body weight, were assigned randomly to two experimental groups: (1) mice fed standard diet (SD—6.55% kcal from fat and 3.9 kcal/g; 4RF21, Mucedola, Milan, Italy) and (2) mice fed a custom pellet high fat diet (HFD—45% kcal from fat and 5.2 kcal/g manufactured by Mucedola) with a modified SD formula. Thus, we obtained four experimental groups: Npy1r*^rfb^* mice on either SD (*n* = 8) or HFD (*n* = 9) and Npy1r*^2lox^* mice on either SD (*n* = 11) or HFD (*n* = 12). Throughout the study, food and water were available ad libitum to all experimental mice. Figure 1 shows the experimental outline.

#### 4.5.1. Body Weight and Food Intake

During all the procedures, including the 1-week baseline, body weight and food intake were determined from 10 a.m. to 11 a.m. every other day. Pre-weighed food was placed in the U-shaped metallic feed hopper of each cage and then re-weighed on a per-cage basis. Food crumbles with a diameter greater than 1 mm were recovered in the cage and weighed as well. Food intake was quantified by calculating the difference between pre- and post-weighed food for the entire duration of the experiment and averaged over baseline and weekly. Food intake was transformed to kcal and expressed as fold changes vs. baseline.

#### 4.5.2. Home Cage Locomotor Activity

Individual daily activity was assessed by means of an automated system that uses small passive infrared sensors positioned on the top of each cage (ActiMeter, TechnoSmart, Rome, Italy) as previously described [55]. Locomotor activity was continuously monitored throughout the whole experiment including one week of baseline phase and 26 days of diet exposure. Recording was interrupted only during the daily manipulations of mice by the operator. 

#### 4.5.3. Glucose Tolerance Test (GTT)

On the 21st day of diet, GTT was performed following an overnight fasting (12 h). Blood glucose levels from tail bleeding were monitored at 0, 30, 60 and 120 min after i.p. injection of a 1 g/kg d-glucose solution (Carlo Erba Reagent S.R.L., Milan, Italy). Blood glucose concentration was determined through glucose meter Accucheck Aviva (Roche Diagnostics, Indianapolis, IN, USA) during the light phase from 9 a.m. to 11 a.m. Mice were then returned to their diet regimen for 5 days to allow the body weight recovery.

#### 4.5.4. Blood and Organ Collection

Five days after the GTT test (day 26) and following 12 h of fasting, mice were euthanized by decapitation after a brief CO_2_ exposure. Trunk blood was collected in heparinized tubes (Sarstedt, Nümbrecht, Germany), centrifuged at 4000 RPM, 4 °C for 10 min and stored at −20 °C until analysis. Visceral white adipose tissue (perigonadal, mesenteric, retroperitoneal, perirenal fat pads) was collected and weighed, then frozen in liquid nitrogen for later analysis. Brains were removed and immediately frozen in liquid nitrogen, then stored at −80 °C until analysis.

#### 4.5.5. Plasma Analysis

Plasma triglycerides, LDL, HDL and total cholesterol were measured on a clinical chemistry analyzer (HITACHI 911, Roche Diagnostic, Indianapolis, IN, USA) using specific Sentinel KIT. Level of circulating corticosterone was measured in Npy1r*^rfb^* mice on either SD (*n* = 5) or HFD (*n* = 5) and Npy1r*^2lox^* mice on either SD (*n* = 5) or HFD (*n* = 5) in duplicate with a commercially available RIA kit (Diagnostic Systems Laboratories, Inc., Webster, TX, USA) with a sensitivity of 0.06 ng/mL. The intra-assay variability was 3.4%. To avoid the interassay variability, all samples were run in a single assay.

### 4.6. In Situ Hybridization for Npy1r mRNA

Non-radioactive in situ hybridization was performed on coronal sections (14-µm-thick) of Npy1r*^rfb^* mice fed SD (*n* = 6) and HFD (*n* = 7) and of Npy1r*^2lox^* mice fed SD (*n* = 8) and HFD (*n* = 8), as previously described (Longo et al. 2018). *Npy1r* mouse probes (489 bp) were obtained by PCR on cDNA using the following primers: Y1R-FW: TTCTCCCTCCAGTGACACTC; Y1R-RV: GGAGACACATGACCGCAAC. For each mouse, six to ten sections spanning from: −1.58 and −1.82 mm relative to mouse Bregma for the arcuate nucleus; −0.82 mm relative to mouse Bregma for the PVN; −1.46 mm relative to mouse Bregma for the medial amygdala; −1.70 and −1.94 mm relative to mouse Bregma for the hippocampus; −1.70 mm relative to mouse Bregma for the dorsomedial nucleus; −1.58 and −1.70 mm relative to mouse Bregma for the basolateral amygdala were used to measure *Npy1r* mRNA relative levels.

The analysis was performed with ImageJ software (NIH). The area of interest of clearly distinguishable nuclei was defined following the boundaries of the labeled regions. Optical densities were measured and averaged after a rodboard calibration. Background was measured by averaging three to five spots [optical density (OD) unit] in the surrounding blank region of the nucleus to be evaluated, then subtracted from the corresponding nucleus value.

### 4.7. Statistical Analysis

Maternal behavior exhibited by CD1 foster mothers was analyzed by Wilcoxon signed rank test. A one-way ANOVA (genotype) for repeated measures was run to analyze body weight growth from weaning to adulthood. A two-way ANOVA (genotype and diet) for repeated measures (days) was run to analyze body weight, food intake and glucose tolerance test during metabolic challenge. A two-way ANOVA (genotype and diet) was run to analyze abdominal adipose tissue, plasma and *Npy1r* mRNA expression. Planned comparisons of Npy1r*^rfb^* and Npy1r*^2lox^* mice from each diet regimen (SD or HFD) and planned same-genotype comparisons across diet and time were assessed by ANOVA and t-test. Data were analyzed by means of Statistica 10.0 (Stat-Soft, Tulsa, OK, USA). The significance level was set at *p* < 0.05. Post hoc tests were performed by Tukey test.

## 5. Conclusions

Limitations of the study:

- No data were collected on the components of energy metabolism (e.g., energy expenditure and respiratory exchange ratio).
- Anxiety-like behavior was not measured in mice used in this study.
- Only males were used in this study, their female siblings were used in a different parallel study (Paterlini et al. in preparation).

Highlights of the study:

- Limbic NPY-Y1R system is involved in energy balance and emotional behavior.
- High fat diet increases body weight, food and calory intake, glycemia and fat depots in male mice with limbic Y1R gene deletion.
- Limbic *Npy1r* gene deletion increases susceptibility to diet-induced obesity in male mice.
- Selective inactivation of limbic *NPY1r* gene might be involved in emotional eating disorders.

## Figures and Tables

**Table 1:** Plasma level of total cholesterol, LDL, HDL and tryglicerides.

Genotype	Cholesterol (mg/dL)		Cholesterol LDL (mg/dL)		Cholesterol HDL (mg/dL)		Triglycerides (mg/dL)	
	SD	HFD	SD	HFD	SD	HFD	SD	HFD
Npy1r*^2lox^*	115.25 ± 6.06	157.45 ± 7.71 *^#^*^#^	6.63 ± 1.05	10.89 ± 1.25 ^†^	62.75 ± 3.38	75.56 ± 3.31 ^†^	71.50 ± 3.76	83.34 ± 11.47
Npy1r*^rfb^*	100.43 ± 5.49	160.75 ± 10.04 ^§§§^	5.43 ± 0.37	11.63 ± 1.52 ^§^	54.00 ± 3.16	76.75 ± 4.69 ^§§^	76.86 ± 9.21	82.88 ± 8.93

^§^*p* < 0.05 versus Npy1r*^rfb^* on SD; ^§§^ *p* < 0.01 versus Npy1r*^rfb^* on SD; ^§§§^ *p* < 0.001 versus Npy1r*^rfb^* on SD; ^##^ *p* < 0.01 versus Npy1r*^2lox^* on SD; ^†^ indicates a trend to significance versus Npy1r*^2lox^* on SD (LDL, *p* = 0.061; HDL, *p* = 0.079).

## Abbreviations

Arc	arcuate hypothalamic nucleus
AUC	area under the curve
BLA	basolateral amygdala
CA1	cornu ammonis 1 hippocampal subregion
CA3	cornu ammonis 3 hippocampal subregion
CamKII	calcium/calmodulin dependent protein kinase II
Cre	cre recombinase
DM	dorsomedial hypothalamic nucleus
DG	dentate gyrus
HFD	high fat diet
MeA	medial amygdala
NPY	neuropeptide Y
*Npy1r*	gene encoding the Y1 receptor for NPY
PND	postnatal day
PVN	paraventricular hypothalamic nucleus
SD	standard diet
Y1R	Y1 receptor
